# The political economy of health in conflict: Lessons learned from three states in the Eastern Mediterranean Region during COVID-19

**DOI:** 10.7189/jogh.12.07001

**Published:** 2022-02-12

**Authors:** Fouad M Fouad, Lurdes Soares, Jasmin Lilian Diab, Alaa Abouzeid

**Affiliations:** 1 Refugee Health Program, Global Health Institute, Faculty of Health Sciences, American University of Beirut, Beirut, Lebanon; 2 Independent researcher, Porto, Portugal; 3 Institute for Migration Studies, School of Arts and Sciences, Lebanese American University, Beirut, Lebanon; 4 Department of Social and Education Sciences, Lebanese American University, Beirut, Lebanon; 5 Faculty of Medicine, Cairo University, Cairo, Egypt; 6 Operational Partnerships, World Health Organization Regional Office for the Eastern Mediterranean, Cairo, Egypt

## Abstract

**Background:**

The Eastern Mediterranean Region continues to face a severe scale of emergencies as a direct result of conflict and political instability in a number of countries. As of 2020, nine countries out of 22 countries in the region affected by protracted and ongoing wars and conflict, left more than 62 million people in dire need of access to quality health care and adequate response measures. COVID-19 exacerbates the humanitarian needs of the people especially in countries that suffer from humanitarian crises, and drains the already overstretched health care systems. This study was conducted to derive major takeaways and lessons learned from the COVID-19 response in humanitarian and low resource settings that may assist similar vulnerable and fragile settings in different regions in view of a possible next pandemic.

**Methods:**

The study involved a desk review, document analysis, and key informant interviews with key stakeholders from the Eastern Mediterranean Region.

**Results:**

A total of 35 key informant interviews were carried out with health professionals working in humanitarian and low resource settings in the region. This study focuses on the information gathered from Afghanistan, Iraq and Syria.

**Conclusions:**

A key finding of this study is that each of the nine pillars for COVID response has been implemented differently across the different countries. Although the nine pillars guide the overall response to COVID-19 in the region, they also provide countries with an important starting point and an important implementation tool.

A strong preparedness guided by a preparedness plan that is adjustable and able to integrate new realities is of utmost importance to an effective response. This needs to be the central role of any government in its COVID-19 response. In addition, it is important for the government to play a key role in the inclusion and coordination of various stakeholders and efforts on the ground.

The first confirmed case of COVID-19 in the Eastern Mediterranean Region (EMR) was in January 2020 in the United Arab Emirates (UAE) [1]. The majority of the countries in the region witnessed slow transmission of COVID-19 during the first few months of the pandemic; however, cases increased during the second half of August 2020.[1] A number of regional responses to COVID-19 specific to the EMR can inform more effective disease control. One of the most effective approaches witnessed throughout the region was the early implementation of public health and social measures beginning in mid-March 2020, including non-pharmaceutical individual and societal interventions to control COVID-19 [1]. Detecting and responding to the COVID-19 pandemic in the EMR currently constitutes the region's main public health priority. Each of the states in the region have set up mechanisms intended to enhance surveillance systems for early detection, isolation and laboratory confirmation of suspected cases [2]. National COVID-19 surveillance and testing guidelines have been developed in close alignment with, or adapted from, World Health Organization guidelines, and diverse responses and contextualized policies have been developed across the region while tailoring each of the WHO's nine pillars of response to meet local realities and contexts.

The EMR continues to face a severe scale of emergencies as a direct result of conflict and political instability in a number of countries. As of 2020, nine countries out of 22 countries in the region affected by protracted and ongoing wars and conflict, left more than 62 million people in dire need of access to quality health care and adequate response measures [3]. COVID-19 exacerbates the humanitarian needs of the people especially in countries that suffer from humanitarian crises, and drains the already overstretched health care systems. Already weakened health systems are faced with shortages in health care workers, medical supplies, essential medicines, and vaccines - all contributing to the deterioration in the provision of health services. Protracted instability and armed conflict in a number of countries in the region has resulted in increased numbers of trauma care injuries, as well as increased risk of infectious disease outbreaks and lack of essential health care services.

To respond to the recommendations of the Global Health Cluster COVID-19 Task Team study, which explored the challenges Health Clusters and Health Cluster partners face to respond to COVID-19 in humanitarian and low resource settings [4], the present study was conducted by the research group with the intention of highlighting major takeaways from the COVID-19 response, and guiding what successes may be replicated in similar vulnerable and fragile settings in different regions in view of a possible next pandemic. Thirty-five key informant interviews were conducted with experts from stakeholder organizations from 9 of 22 states impacted by humanitarian crises in the EMR through a pre-determined set of open-ended questions to derive some of the good practices, lessons learned and future steps for health care in the region during COVID-19. Of the nine countries that originally took part in the study, this paper delves into three: Afghanistan, Iraq and Syria, with the aim of exploring the contributing or hindering factors to the COVID-19 response in each of these countries. The paper will address political, economic, cultural and social paradigms in order to frame the response in each of these countries, while also highlighting major takeaways from the COVID-19 experience that may inform responses to potential pandemics in the future in fragile, vulnerable and conflict-affected settings.

## METHODS

The study involved a desk review, document analysis, and key informant interviews with key stakeholders from the EMR. It addressed the nine pillars of the COVID-19 response as key considerations in developing a strategic approach to quality action planning in such settings as per the WHO’s “COVID-19 Strategic Preparedness and Response Plan”. A set of interview questions were developed by the lead investigator for health clusters and their partners in both English and Arabic to encompass the nine interdependent elements and address contextual needs based on country and conflict situation. A qualitative approach, desk review and document analysis was employed to understand: (1) the health needs/priorities, technical gaps and the operational challenges experienced in the EMR in fragile, conflict-affected and vulnerable (FCV) settings; (2) how priorities and challenges have been addressed. The research team interviewed and gathered in-depth data on perceptions and experiences from Health Cluster Coordinators, WHO Technical Focal Points, International Non-governmental Organizations (INGOs), Local Non-governmental Organizations (NGOs), Ministry of Health partners, and UN agencies who directly provide health and health-related services for the COVID-19 response in FCV settings in the region. This study employs a comparative analysis approach to analyze the raw data.

### Key informant interviews

A total of 35 key informant interviews were carried out by the authors with health professionals working in FVC settings in the region. For this study, only the information gathered from Afghanistan, Iraq and Syria is presented.

The lead investigator and two research assistants (the authors) performed the interviews. Despite having experience in this area, the research assistants received training and were supervised by the lead investigator during the initial interviews. A standard interview guide was followed, and a note taker was present in all interviews. The language used was English, except for two participants who preferred to speak Arabic.

Interviews were done remotely, using MS Teams and Skype, and were recorded using the same platforms with the consent of the respondents. Data were stored in a password-protected device and only accessible to the research team.

Prior to analysis, an edited transcription of all the recordings was done and the interviews in Arabic were translated into English.

The data was analyzed qualitatively through the steps of (1) identifying biases and noting overall impressions, (2) reducing and coding data into emerging central themes, (3) searching for patterns and interconnections that can inform potential areas of programming, (4) mapping and building emerging themes and areas of concern, (5) building and verifying theories, and (6) drawing conclusions, lessons learned and providing recommendations. All interviews were conducted in accordance with best ethical practice in research, particularly with respect to ensuring participants’ safety, anonymity (if/when requested), the protection of data, and risk mitigation. The study additionally followed the Humanitarian Principles of humanity, neutrality, impartiality and operational independence.

## RESULTS

In the EMR, nine of twenty-two states are impacted by humanitarian emergencies, many of which are an immediate result of war, political and economic instability, man-made or natural disasters [4]. Key informants point to the fact that these emergencies have considerably debilitated or disrupted health systems in multiple states in the EMR – as is the case in Afghanistan, Iraq and Syria. Fragile and vulnerable groups constitute a sizeable population in these states. Accordingly, the region retains 43% of those in need of international aid and humanitarian assistance and outsources 64% of the world's refugees [5]. The EMR additionally retains one third of the world's IDPs [5]. These particularly vulnerable populations are at heightened risk due to poor living conditions, and many remain ostracized with limited access to necessary and adequate health care services. Economic barriers make this access difficult as well. In the context of COVID-19 and its spread across the region, refugees, IDPs and populations in need in emergency settings continue to experience instability and increased risk.

### Additional strains on an already fragile health system in Iraq

Although Iraq has gradually begun to recover from the several waves of protracted conflicts and humanitarian emergencies it has experienced these past decades, the socio-economic impacts of COVID-19 have exposed the country's population to additional challenges, perpetuated existing vulnerabilities and placed additional pressure on an already fragile public sector [6]. With petroleum accounting for the majority of the country's exports (close to 92%), declining oil prices have plunged Iraq into an economic crisis, with the World Bank predicting a reduction of the Iraqi GDP by 9.7% in 2020 [7]. As the country continues to grapple to meet the salaries of its six million public sector employees, millions of employees from the private and informal sector have lost their employment as a result of the COVID-19 crisis [7]. UNICEF and the World Bank found that four million Iraqis fell below the poverty line in 2020 [7]. With this drastic increase in unemployment and poverty rates, humanitarian needs across all sectors and in the most vulnerable factions of the population increased drastically in 2020 – which ultimately imposed additional strains on Iraq's 1.4 million Internally Displaced Persons (IDPs), close to 5 million returnees and 286 000 refugees and asylum seekers [8].

*Country-level Coordination, Planning, and Monitoring (CCPM)* structures were agreed at the early stages of the pandemic, effectively assisting Iraq in mounting the national response. From the outset, the WHO played a crucial role, providing technical and strategic guidance. The Humanitarian Country Team, the UN Country Team, health cluster and other clusters, government institutions, all relied on the WHO for decision-making and strategic guidance to manage the COVID response in the country. Everything from WASH to socio-economic impacts of COVID-19 were addressed under coordinated structures and were communicated to the Ministry of Health early on. A clear coordination mechanism that works in the fragile settings in Iraq proved to be pivotal to the success of this pillar. The Iraqi national level response was highly swift and effective with curfews, social distancing measures taken, closure of restaurants and public places all over Iraq since March 2020 [9]. These measures took place in close coordination with stakeholders to ensure that community transmission did not surge. While imposing restrictions and preventive measures in Iraq was a particular challenge when it came to the camps, early stages of coordination largely contained transmission, and proved to be pivotal when addressing the other pillars in the response plan [9].

Ongoing *Communication and Community Engagement (RCCE)* efforts were heavily complemented with communication plans and the integration of accessible media tools that catered to FVC settings across the country. In areas where access to connected devices was limited, radio messages, billboard messages and SMS messages were used as the “right means of communication” in targeting specific groups such as refugees, IDPs and people from ongoing conflict settings. Through this bottom up approach, stakeholders in Iraq largely succeeded in ensuring a comprehensive and integrated approach in this pillar of their response.

Though Iraq has managed to implement the aforementioned pillars, the country witnessed less success in *Surveillance, Rapid Response Teams, and Case Investigation*, where the government surveillance system “did not hit the mark”. Surveillance efforts in this area were hindered by weak government surveillance systems. The country heavily relies on the WHO's Early Warning Alert and Response Network (EWARN) – a network of health partners that collect and report surveillance data on selected epidemic-prone diseases, as part of establishing an early warning system for disease outbreaks in humanitarian situations. Although this is regarded as a functional tablet-based reporting system that focuses on 16 priority diseases, it only operates in locations that are supported by health cluster partners (according to a key informant, this covers about seven or eight governorates where health partners are providing humanitarian health services). The Health Cluster continues to work closely with the government to integrate this into the regular surveillance system of the Iraqi government.

Stakeholders on the ground and the health cluster have proposed an innovative intervention that permits them to conduct the needed testing in their Primary Health Care Centers (PHCC). This model collects the testing samples from inside the camps, stores in accordance with official guidelines of sample safekeeping, and sends them for testing at the Department of Health (DoH). This, according to an expert from Iraq, constituted “a great relief and a great help”.

UN Agencies have played a pivotal role in training local health staff in Iraq. Organizations such as the WHO and UNICEF, effectively identified key focal points from within the Ministry of Health’s laboratory departments. These focal points were trained on COVID-19 testing, surveillance and management in order to serve as the support these UN agencies needed in camp settings, and among vulnerable and fragile groups. This approach encompassed what experts referred to as “refresher trainings” by UN Agencies - retraining taken by a person already qualified or previously assessed as competent in a field with the intention of updating skills and/or knowledge to a changed standard, or providing the opportunity to ensure that no important skills or knowledge have been lost due to lack of use. This model was also adapted to meet COVID-19 restrictions and the inability to conduct the training sessions in person. They were adapted into an online model to reach out to 100-150 people per training session. These trainings have been given in Iraq to staff from the Ministry of Health, laboratory focal persons and for surveillance focal persons. In terms of providing them with testing kits and personal protective equipment (PPE), multiple obstacles presented themselves in procurement and logistics. While this was combatted through working closely with local governments who are tasked with doing a large share of this distribution themselves in many countries in the region, this form of coordination in the areas of procurement and logistics did not prove to be adequate in Iraq. In many cases, according to an expert from Iraq, there were issues with the rapid diagnostic tests. Whilst the Iraqi government was trying to use them to diagnose current cases, the use of rapid diagnostic tests according to an interviewed expert is “against everything that we preach as WHO”. The WHO is working closely with the Iraqi government to use PCR tests for case detection.

The three states included in this paper have all conducted an IPC assessment during COVID-19 response. In Iraq, this assisted in placing *Infection, Prevention and Control* on the list of priorities of hospitals – as this was not practiced prior to COVID-19. This practice additionally assisted in making IPC an ongoing focus for the Ministry of Public Health and health workers in countries such as Afghanistan and Iraq. To move forward in this area in fragile and vulnerable settings, health clusters in Iraq trained their own staff in IPC, the disinfection of public places, and in strengthening hygiene promotion activities.

### A solid response in Afghanistan

As a donor-dependent conflict-affected country, Afghanistan struggles with health care delivery and navigating through limited health literacy and preventative measures, shortages of skilled health workers, and fragile health infrastructure [10]. In Afghanistan, 75% of the population live in the rural areas and 80% live below the poverty line [11]. Given the country's close proximity to China, Afghanistan's Ministry of Public Health (MoPH) began discussing preparedness measures for COVID-19 as early as December 2019 and anticipated that more than 80% of the population could ultimately be infected if preventative measures were not followed [11]. Since the outbreak of the COVID-19 pandemic in Afghanistan, the WHO has worked closely with the country's MoPH to ensure that the COVID-19 Strategic Preparedness and Response plan's nine pillars were adequately upheld [12]. This WHO-led partnership has ensured the laboratories' readiness, has trained technical and medical personnel, as well as provided equipment and supplies to broaden testing to vulnerable groups throughout the country [13]. In Afghanistan, over 2000 health care personnel were trained on case management and intensive care, and more than 1300 received training on IPC through WHO [13].

Afghanistan's disease early warning system and its existing polio program's extensive surveillance network significantly assisted preparedness for the COVID-19 response, and gave the country a strong advantage over other vulnerable states in the region [14]. In an effort to assist the most vulnerable communities within the country, Afghanistan has additionally channeled great effort on increasing cross-border collaboration between its neighboring countries to manage COVID-19 [14]. The country has further developed – in close collaboration with WHO – a strategic vision for risk communication and community engagement (RCCE) for inclusion in the country's existing national action plan [14]. The country's strategic partnerships with international bodies such as the World Bank have further cemented the country's successful responses. The World Bank’s support to the country, coupled with the government's cooperation, has improved water supply, sanitation, and hygiene as part of the COVID-19 response [15]. Afghanistan has additionally been working closely with the World Bank to ensure financial intermediation so that micro, small and medium enterprises can have access to finance, and to expand the use of mobile money throughout the country [15].

*Country-level Coordination, Planning, and Monitoring (CCPM)* efforts in Afghanistan since the beginning of the COVID-19 pandemic laid a solid foundation for the establishment of the other pillars in many cases. UN agencies, humanitarian country teams and international organizations operating on the ground have coordinated among themselves as well as with the government to ensure the success of the response. In Afghanistan, as highlighted from interviews with experts working on the ground, there is a unified UN-COVID-19 Response Plan that each UN agency contributes to under the leadership of WHO [16]. The health cluster has additionally ensured adequate implementation of necessary measures through its COVID-19 multi-sectoral response plan. Afghanistan’s success in CCPM has been attributed to the centralized leadership under WHO – whereby WHO leads on a group of UN agencies each working on pillars under their mandates and areas of expertise. For example, when addressing *Points of Entry* (PoE), IOM takes the lead while also reporting back to the WHO in order to centralize the response and avoid any duplication of work, or any mutually exclusive work [17]. The IOM Displacement Tracking Matrix (DTM) collects information weekly about current COVID-19 movement restrictions at points of entry in Afghanistan in coordination with WHO and the government since March 2020 [17]. In the words of a key informant, *“the coordination in leading the whole COVID-19 response is a good practice. (…) When coordination structures were clear, people knew exactly where to go.”*

Initiatives such as mapping of stakeholders by Ministries of Health, while ensuring that services are not duplicated across different actors, played an essential role in improving coordination, planning and monitoring. In Afghanistan, this serves as a clear illustration of coordination labors to avoid overlap and duplication of efforts. In Kabul, Johanniter International (JOIN) and CARE International both implemented a COVID-19 response [18]. In a meeting with the Provincial Public Health Directorate of Kabul each stakeholder identified the areas where they will be working and additionally coordinated with different partners in the areas of WASH and shielding practices linked to COVID-19. While progress is being achieved in coordination, planning and monitoring, improvement is recommended in planning by the Ministry of Public Health to assume a leadership position in managing stakeholders and different actors on the ground.

Working closely on the community level has proven to be pivotal when it comes to communicating trusted messages about COVID-19 to the general public, specific constituencies and vulnerable groups. In settings where groups such as women, individuals living in inaccessible areas, people with disabilities, as well as other groups like IDPs and refugees, do not have access to that same level of mass media information, reaching out to community figures showed effectiveness in Afghanistan, according to a key informant. Working with grassroots organizations, community leaders, elders, religious leaders, and prominent members of the community have not only assisted stakeholders in identifying specific needs and approaches, but have also assisted in communicating information to these communities that they trust. Working with community leaders through a community-based approach proved to be essential in terms of awareness raising. Through this approach, community leaders have additionally been successfully trained on risk communication and community engagement in order to ensure that they are communicating the correct information to the people in their communities. This has assisted in combatting rumors and false information, as well as in reducing panic among communities across the country.

The importance of public and private partnerships was repeatedly highlighted throughout the fieldwork for this study as one of the major contributing factors to the implementation of the *Surveillance, Rapid Response Teams, and Case Investigation* pillar. Initially, in Afghanistan, the government only allowed for government health facilities to do the testing. With the spread of COVID-19, government health facilities opted for assistance from the private hospitals and clinics once they were over capacitated. Even in the area of treatment and isolation, partnerships between the public and private sectors have constituted an essential good practice from Afghanistan. Similar to Iraq, the COVID-19 response in Afghanistan assisted in placing *Infection Prevention and Control (IPC)* on the list of priorities of hospitals, and in making IPC an ongoing focus for the Ministry of Public Health and health workers. To move forward in the areas of IPC in fragile and vulnerable settings, the health cluster in Afghanistan also trained health care workers in IPC, the disinfection of public places, and in strengthening hygiene promotion activities.

### A struggle with case management in Syria

A decade of ongoing conflict has devastated Syria's health system and displaced over 70% of health care workers [19]. Exacerbating this pre-existing humanitarian crisis is COVID-19. As per Key Informant Interviews, the true extent of the COVID-19 outbreak remains unknown due to limited testing capacity, underreporting, lack of access to adequate health care services among other factors [19]. Destruction of health facilities, the displacement, migration and death of health care workers, and fractured health systems have worsened the humanitarian situation and severely hindered local and national capacities and services [19]. Even prior to the pandemic, Syria's fragmented and incapacitated health system was unable to cope with the scale of health emergencies, and did not have the capacity to adequately respond to outbreaks and epidemics, such as polio and cholera [19]. The protracted conflict has led to a fractured health system, with government-controlled areas, the northwest, and the northeast each having distinct systems of governance and corresponding humanitarian responses [20]. There are close to 10 million people in government-controlled areas, 4.2 million in the northwest, 2.7 million in the northeast, and an additional 5.6 million Syrian refugees in neighboring countries [20]. Due to the decentralized nature of the present Syrian health system, NGOs and local health directorates have provided services and overseen all health campaigns in coordination with the national health system where possible [20].

While adequately tailoring the response to meet the WHO nine pillars in countries as fragile as Syria, a particular obstacle presents itself in the form of *Case Management* according to experts of each of Syria's three regions. While it has proven to be one of the main obstacles in the region as a whole (as multiple states in the region lack the functional surveillance systems that can uphold this pillar), in Syria the lack of a functional surveillance system has proven to be a hindrance to effective case detection, effective referral, and to ensuring that case management facilities are utilized in order to identify most vulnerable people. The lack of functional surveillance systems for the pandemic in Syria is mostly attributed to the fact that there was no independent surveillance system in place prior to COVID-19 in the country. Some of the obstacles in this regard have been the lack of transparency, the absence of a feedback mechanism, the lack of access to information, and the lack of trust among partners. The lack of coverage is yet another challenge in the area of surveillance. To combat this, the WHO has been working on an autonomous surveillance capacity with other partners. According to an expert working in Syria in the Idlib region, they have developed a rudimentary surveillance system through the hotline from scratch, in order to ensure that they have visibility on samples collected from Northeast Syria which were going to Damascus for testing – but this model has not been effective.

## DISCUSSION AND LESSONS LEARNED

While responses across the EMR have produced patterns and models that may work in different contexts, much of the responses are context-specific and tailored to escalating situations of vulnerability, fragility and decades long protracted crises. The study has found that close coordination between the Health Cluster, UN agencies and Ministries of Public Health constituted a major component of the response throughout the EMR – although the level and integration of this coordination varied along the phases of the pandemic response. In Afghanistan for instance, implementation modalities included the deployment of mobile teams in different geographical locations, with mobile health teams having different levels of intervention. While coordination was primarily done through working directly with communities through mobile health teams, and second through building capacities of existing staff in local health facilities to complement these teams and be present on the ground, there remained much more room for improvement in training and capacity building efforts for these teams and communities to ensure that they delivered accurate information and did not hinder any of the overall goals of the response. Innovative approaches that involved working closely with community, provincial and religious leaders, as well as community representatives, teachers, community health workers, and prominent members of society to reach out to particular communities from a relatable source did prove resourceful and helpful throughout the response. However, this approach did not prove as effective when it came to the manner through which information was presented to particular factions of society – as the rapid development in information pertaining to the virus was not always adequately and “equally” delivered simultaneously.

In Iraq, INGOs such as the International Medical Corps (IMC) have continued to support refugee camps with primary health services [21], and have requested extra funding to maintain a solid and effective COVID-19 response and containment strategy. One of the main goals for states with protracted crises and vulnerable populations was to keep facilities running throughout the crisis, and assist as many people as possible in order to avoid community transmission in highly densely populated areas such as camps. However, efforts in this regard were challenged by multiple realities on the ground including a lack of infrastructure in these settings, a lack of compliance among members of these groups, close quarters in these areas as well as one's inability to isolate or quarantine – and to a large extent, these realities cannot be avoided or comprehensively addressed. In Iraq, IMC worked closely with these communities to separate the cases between respiratory and non-respiratory cases (in other words suspected COVID-19 and non-suspected COVID19- cases). They used what they call a ”surge staff” to support their work on the COVID-19 response. This allowed for the differentiation of the cases, and examination by especially dedicated staff that are more trained and more equipped to deal with COVID-suspected patients according to the latest WHO/Ministry of Health guidelines [21].

Some of the most effective response models encompass a reliance on previous experiences tackling health crises as well as on existing structures. In Afghanistan, Iraq and Syria where similar previous experiences have not been encountered, this largely meant there was a need to tap into innovative and intersectional approaches to the response. INGOs and UN agencies across all three countries have had to maintain the delicate balance between the response and the prevention components of tackling COVID-19. Working jointly across UN agencies has also served as a determining factor in the success of the response, as WHO collaborates with IOM, UNHCR, and UN Women and others in the coordination of the response, secondary outcomes and shielding practices.

Reliance on an already-existing “crisis management” response system has aided in addressing COVID-19 in some of the region's most fragile states. In the case of Syria, where economic collapse and shortage of resources, medications, consumables and laboratory materials have been the norm since the outbreak of the Syrian conflict one decade ago, coordination among UN and humanitarian agencies to provide primary, secondary and some tertiary care was already regular practice. Amid COVID-19, and with an overstretched, fragile system, WHO is the health cluster lead agency that brings together partners that are operating across all three regions, and adapts health services to populations who are mainly cut off from accessing Syria's different regions.

In theory, it is the Ministries of Health that lead the response and coordinate efforts to address the situation, however some of the region's most vulnerable states have heavily relied on the existence of international humanitarian organizations to work through an adequate response plan. In Syria, although the country's health system was pre-dispositioned to work in states of emergency and crisis as stated previously, it currently works closely with a number of international organizations and UN agencies, and operates under the close supervision of the health cluster. By the end of 2019, the GoS was in control of around 62% of the Syrian territory [22]. With no operational government agencies due to the fragmentation of power, the country's Health Cluster Co-coordinator, the International Development Association (IDA), works specifically on *points of entry*. They have teams on the checkpoints to screen the travelers and returnees crossing the border, and detect suspected cases to be referred to COVID-19 dedicated health facilities.

Though the health clusters and the WHO are heavily present within each of the aforementioned EMR states, responses have fallen victim of over-shadowing humanitarian crises that have heavily hindered the establishment of a comprehensive response in many of the region's most fragile states. This has led to the limiting of the response to a few pillars under the cluster due to the severity of the conflict and immediate and escalating needs. The COVID-19 response has fallen short in addressing any pre-existing crises, and in ensuring that the secondary outcomes of the virus would not further exacerbate the country's vulnerability.

One of the major lessons learned from the COVID-19 experience in the three countries was the importance of utilizing existing structures in terms of the humanitarian actors in order to maximize the success of the response. While government bodies and Ministries of Health have a pivotal leadership role to play, one of the most important components of the response remained the utilization of the resources present in each country (UN Agencies, INGOs, local NGOs and other expert stakeholders) in order to reach vulnerable and fragile groups in conflict settings – particularly IDPs, refugees and asylum seekers. As the case of Iraq has informed the study, some of the most successful responses to COVID-19 carried out involved many of the non-health actors, protection clusters and NGOs who were capacitated to deliver information at the community level to the communities they had already been serving. In Afghanistan, and according to the informants, the approach employed for RCCE proved to be effective, mainly due to the involvement of non-health actors from the NGO field. Although the WHO takes the lead in RCCE for COVID-19 in the country, RCCE working groups for COVID under WHO's umbrella have been established and co-chaired by the Norwegian Refugee Council (NRC), which is a protection NGO [23]. In this model, working groups include a variety of different actors in the areas of health and protection in order to disseminate the information, raise awareness and combat misinformation.

Another lesson learned lies in the importance of initial surveillance and testing of people across all communities and groups within a given population – whether this be nationals, migrants, refugees, or IDPs. Through a rapid response approach, people can be easily and quickly identified in contact tracing. Establishing a strong foundation in the area of surveillance and testing will allow for shifting the focus to the more basic components of treatment such as securing ventilators, PPE and other necessary consumables. This still needs to be heavily addressed in Syria, where different governing entities continue to navigate across different regions and different responses.

Early on in the response of the pandemic, stakeholders and organizations acting on the ground learned the importance of having a strong centralized mechanism that coordinates all efforts and activities in the COVID-19 response. This is important not only in order to recognize where partnerships and networks can be established within the response, but also in order to avoid the duplication of efforts among various target groups and communities. It is important that governments involve all stakeholders so they can support the government and the strategy early on. Moreover, an early preparatory plan outlined by the Ministry of Public Health must be consistently updated and communicated to stakeholders to encompass developments and new information as it rolls in; it should also be amended with the increase in cases as they appear. One of the major lessons learned is that preparation and strategy responses to the pandemic outlined by governments should have been prepared earlier – this was a major foundation for the successful responses in countries such as Afghanistan that adopted this approach early on. Particularly, the response for increasing hospital capacity, which was very slow in states across many conflict-affected states in the EMR at the early stages of the pandemic. This could have reduced the need to orient private hospitals in order to increase that capacity on short-notice such as in Iraq. Furthermore, Ministries of Health needed to develop strategic plans and communicate them in the form of unified messages to humanitarian actors sooner – particularly because they work with fragile and vulnerable groups. Plans across the region have been interpreted as more reactionary than pre-emptive in the case of Syria for instance.

Lack of preparedness remained a challenge in countries such as Iraq and Syria, and COVID-19 has shown actors on the ground that they were not prepared prior, and will most likely remain under-prepared for the next pandemic or global health crisis if the structures established in response to COVID-19 are not maintained. While countries such as Iraq collaborated with humanitarian and development actors to swiftly mount a response, challenges presented themselves in other forms. This is mainly due to several key factors that are not likely to be improved or changed over time such as the absence of testing capacity, the absence of adequate testing laboratories, the very limited number of ventilators and obstacles in procurement and import due to government bureaucracies and restrictions. Other factors such as neglecting to uphold International Health Regulations, neglecting incoming people from designated virus hotspots, and the lack of preparedness on the potential spread of disease across borders also constitute contributing factors to this realization.

In contexts such as Syria, where nationwide preparedness proved to be a challenge, actors on the ground quickly learned that there was a degree of preparedness in terms of a similar type of response in the areas of conflict and crisis management that would assist in navigating the pandemic to a certain extent. Experience in the area of swift mobilization and swift provision of health services serves as a solid foundation for the development of a COVID-19 response. Having this solid foundation assisted in diverting attention towards building local NGO capacity, and strengthening their ability to respond to future outbreaks. This solid foundation also assisted in strengthening internal networks even if the response was sidetracked by the protracted ongoing conflict. It also assisted in lessening the blow of having a reactionary approach to the response – as although the response came in “late” (as a key informant explained), the already-existing humanitarian infrastructure assisted the COVID-19 response to a large extent.

The initial phase of the pandemic brought about a reactionary assessment of internal vulnerabilities, and this prevented the development of partnerships at the early stages of the pandemic. In many cases, this was coupled with government have little-to-no resources to fill these gaps in the response strategy. Governments in some of the region's most conflict-affected countries were additionally unable to outline exactly what they need amid struggling with their own administrative obstacles and poor health systems – such as in Iraq and Syria. Because of a focus on internal reality there was a lack of awareness of about the issues of risk – and this impacted the overall response. Prepositioned stock from large INGOs as well as UN agencies present on the ground in Syria assisted in the little success the response had in each of the country's different regions and among some of these regions' most vulnerable groups.

The provision of shelters, quarantine facilities and the ability to practice physical distance constituted one of the major challenges to the success of the response in conflict-affected settings, among vulnerable and fragile groups, and in refugee and IDP settings. While physical distancing sounded like a simple practice in principle, in application it has proven to be one of the most challenging measures to uphold according to a key informant from Iraq. The inability to ensure physical distancing in camps, in crowded hospitals, and in conflict-settings has constituted one of the major drivers of secondary impacts of the pandemic such as gender-based violence, mental health constraints, and the need for shielding practices.

## CONCLUSION

One of the key findings of this study is that each of the nine pillars for COVID response has been implemented differently across the different countries. Although the nine pillars guide the overall response to COVID-19 in the region, they also provide countries with an important starting point and an important implementation tool.

Initiatives that counted on involving local community leaders in the communication strategy of the response, building on existing infrastructures in the country, as well as utilizing their strong local networks to ensure that risk communication and response coordination take place at every level - between UN agencies, stakeholders and government bodies - were fundamental to achieve some level of success in the response to COVID-19. According to the interviewees, other initiatives with respect to capacity building, training of staff, and cooperation between government bodies and UN agencies were also important to deliver tailored approaches to meet the needs of the most vulnerable and fragile groups in conflict settings.

One of the important lessons learned from the COVID-19 experience in the EMR, remains the importance of a strong preparedness guided by a preparedness plan that is adjustable and able to integrate new realities. This needs to be the central role of any government in its COVID-19 response – particularly when multiple stakeholders and government agencies lack the means from proper coordination and communication lines. In addition, it is important for the government to play a key role in the inclusion and coordination of various stakeholders and efforts on the ground.
